# Prosthetic rehabilitation of hypophosphatasia: a case report

**DOI:** 10.1186/1757-1626-2-7626

**Published:** 2008-12-12

**Authors:** Bora Bağiş, Esra Baltacioğlu, Elif Aydoğan, Evşen Tamam

**Affiliations:** 1 Assistant Professor, Department of Prosthodontics, Faculty of Dentistry, Karadeniz Technical UniversityTrabzonTurkey; 2 Assistant Professor, Department of Prosthodontics, Faculty of Dentistry, Karadeniz Technical UniversityTrabzonTurkey; 3 Research Assistant, Department of Periodontology, Faculty of Dentistry, Karadeniz Technical UniversityTrabzonTurkey; 4 DDSPhD, Private Practice, AnkaraTurkey

## Abstract

Hypophosphatasia is a congenital disease characterized by deficiency of serum and tissue non-specific alkaline phosphatase activity. The disease occurs due to mutations in the liver/bone/kidney alkaline phosphatase gene. Six clinical forms of hypophosphatasia are recognized. Systemic symptoms of the disease are respiratory complications, premature craniosynostosis, widespread demineralization and rachitic changes in the metaphases, stress fractures, chondrocalcinosis and osteoarthropathy. Characteristic dental symptoms are premature deciduous teeth loss, premature exfoliation of fully rooted primary teeth, severe dental caries and alveolar bone loss. This clinical report describes the prosthetic rehabilitation of a twenty two year-old Turkish female patient with hypophosphatasia.

## Background

Hypophosphatasia is a rare bone disorder characterized by low or zero levels of the serum and tissue non-specific alkaline phosphatase necessary for normal bone mineralization [1]. The disease occurs due to mutations in the liver/bone/kidney alkaline phosphatase gene encoding the tissue-nonspecific alkaline phosphatase (TNAP or TNSALP) [2,3]. Its function in bone and dental mineralization is still unclear but is connected hydrolysis of PPi [4], collagen [5] and calcium binding [6].

Six clinical types of hypophosphatasia are currently recognized as in the forms of perinatal lethal, benign prenatal, infantile, childhood, adult and odontohypophosphatasia. The prevalence of severe forms of the disease has been estimated at 1/100 000. The incidence of moderate forms was estimated to be much higher [2].

In the lethal perinatal form, the patients have impaired mineralization during intrauterine period. They have skin-covered osteochondral traces on the forearms or legs [7]. Some infants can only live for a few days and have respiratory complications due to hypoplastic lungs and rachitic deformities of the chest.

In the prenatal benign form, despite prenatal symptoms, there is a spontaneous improvement of skeletal defects after intrauterine period [8,9].

Patients with the infantile form may being visible normally at the date of birth; however, the clinical signs of the disease appear during the first six months. Respiratory complications may occur due to rachitic deformities of the chest [10]. Premature craniosynostosis is a common symptom that makes an increase at the intracranial pressure. Hypercalcemia, poor feeding, anorexia, vomiting, hypotonia, polydipsia, polyuria, dehydratation and constipation are the other symptoms. Increased excretion of calcium may lead to renal damage. Premature loss of deciduous teeth is also common dental symptoms of this form [11].

Dolichocephalic skull, enlarged joints, lateness in walking and shortness in length can be seen in the childhood form [12]. Fractures and pain of bone are the usual complaints as well. Premature loss of dentition is common, especially the incisor teeth are the first affected.

The adult form presents during middle age. The first complaint is usually foot pain due to stress fractures of the metatarsals. Osteoarthropathy also may occur later in life. When a detailed history is obtained, premature loss of their deciduous teeth will be common complaint [13,14].

Abnormalities of the skeletal system are uncommon in odontohypophosphatasia. It is characterized by premature exfoliation of fully rooted primary teeth and severe dental caries. The anterior deciduous teeth are more likely to be affected [15]. Reduced alveolar bone, enlarged pulp chambers and root canals can be seen at the radiographic examination. Although the only clinical property is dental disease, biochemical findings are generally same with the other forms of hypophosphatasia. Odontohypophosphatasia should be considered in any patient with a history of early unexplained loss of teeth or abnormally missing teeth are identified on dental examination [11].

Diagnosis of hypophosphatasia is based on clinical and radiographic examinations, laboratory findings (serum alkaline phosphatase activity, PEA and PLP) and molecular biology. Distinctive diagnosis could be done with osteogenesis imperfecta, rickets, achondrogenesis.

This clinical report describes the prosthetic rehabilitation of a twenty two year-old female patient with hypophosphatasia.

## Case presentation

**Figure 1.**
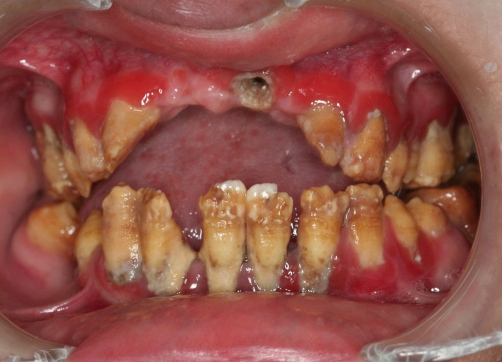
Intraoral view before treatment.

**Figure 2.**
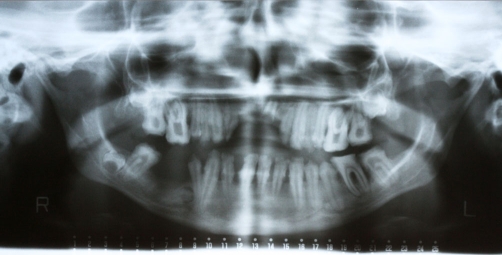
Panoramic radiography.

**Figure 3.**
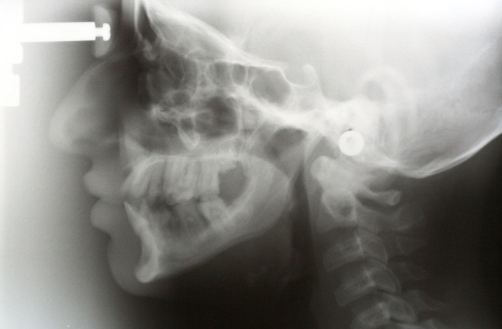
Lateral Cephalometric radiography.

**Figure 4.**
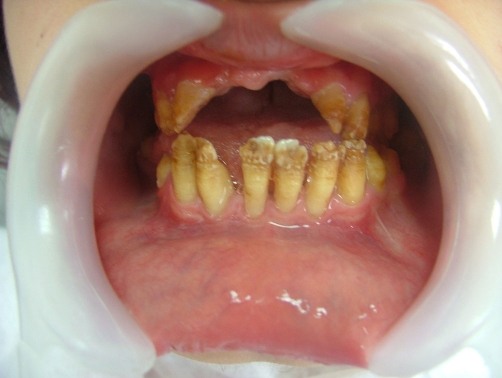
Intraoral view after periodontal treatment.

**Figure 5.**
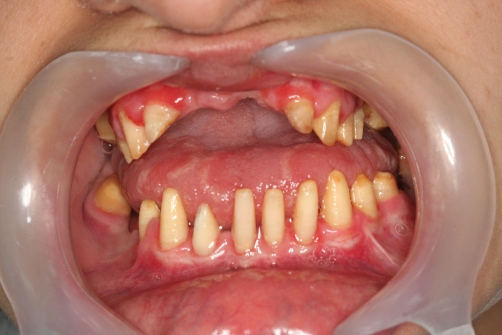
Tooth preparation.

**Figure 6.**
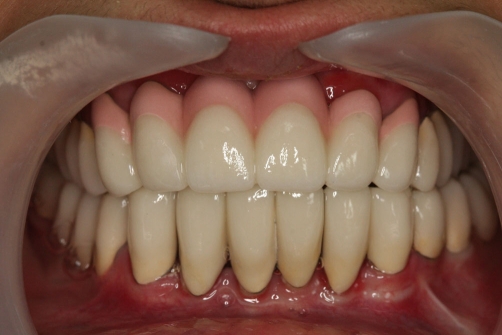
Final restoration.

**Figure 7.**
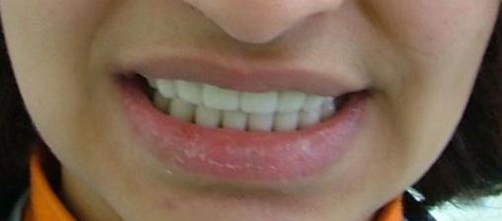
After 3 months control.

A twenty two year-old Turkish female patient with hypophosphatasia was referred to Department of Prosthetic Dentistry in Karadeniz Technical University with poor oral hygiene, missing teeth and unaesthetic appearance. Abnormal enamel formation, dental plaque accumulation and dental caries were detected during intraoral examination (Figure 1). Alveolar bone loss and enlarged pulp chambers were seen in the radiographic examination (Figure 2,3). Radix of maxillary left central incisor and mandibular left first molar were pulled out. Non-surgical periodontal treatment was performed (Figure 4). Oral hygiene was also obtained with the help of her family. Due to the corrupted occlusal relation, teeth loss, patient’s age and motivation, metal ceramic fixed partial denture were decided to construct after periodontal treatment. Topical anaesthesia (Xylocaine Pump Spray 10%, Astra Zeneca, Sweden) was performed for tooth preparation because she never allowed for injection. Teeth were prepared with knife-edge marginal design due to the risk of pulp perforation (Figure 5). Teeth preparations were not enough because patient was anxious and sleepy at the chair time. Impressions were taken with polyvinylsiloxane impression material (Speedex Putty ve Speedex Light Body, Coltene AG Altstatten, Switzerland) after controlling tooth preparation. Metal ceramic restorations were constructed after adaptation. Occlusion was adjusted for a better function and aesthetic appearance was obtained (Figure 6). Provisional cementation was performed with an eugenol free temporary luting agent (Cavex, Temporary Cement, Cavex, Holland). Intraoral control was performed after three weeks and restorations were then cemented with polycarboxilate (Adhesor Carbofine, Spofa Dental, Czech Republic). There was a better oral hygiene in the next third month control and she was pleased with her new appearance (Figure 7). Her motivation of oral hygiene had been increased.

## Discussion

In which form of hypophosphatasia the patient is should be considered before making a dental treatment planning. There are less skeletal deformities but more dental symptoms at the odontohypophosphatasia form. Therefore normal aesthetic and function can be provided with prosthetic rehabilitation. Some difficulties are present during dental applications for these patients. Most important problems are abnormalities of enamel and dentin formation, and enlarged pulp chambers. Several authors have reported that dentin structure has been reduced in thickness and also mineral content of dentin was low in hypophosphatasia [15,16]. One should have been aware of the perforation risk of pulp chamber during tooth preparation. Because of this reason knife-edge marginal design was preferred in this case.

The patient’s anxiety was also a problem during treatment period. Patient didn’t allow for injection and was sleepy during the chair time. These problems were solved by short and effective applications with a team study.

## Abbreviations

PEA and PLP: serum alkaline phosphatase activity
TNAP or TNSALP: tissue-nonspecific alkaline phosphatise
